# Rachis morphology cannot accurately predict the mechanical performance of primary feathers in extant (and therefore fossil) feathered flyers

**DOI:** 10.1098/rsos.160927

**Published:** 2017-02-15

**Authors:** John Lees, Terence Garner, Glen Cooper, Robert Nudds

**Affiliations:** 1 Faculty of Life Sciences, University of Manchester, Manchester M13 9PT, UK; 2 School of Mechanical, Aerospace and Civil Engineering, University of Manchester, Manchester M13 9PL, UK

**Keywords:** birds, primary feathers, structural properties, safety factors

## Abstract

It was previously suggested that the flight ability of feathered fossils could be hypothesized from the diameter of their feather rachises. Central to the idea is the unvalidated assumption that the strength of a primary flight feather (i.e. its material and structural properties) may be consistently calculated from the external diameter of the feather rachis, which is the only dimension that is likely to relate to structural properties available from fossils. Here, using three-point bending tests, the relationship between feather structural properties (maximum bending moment, *M*_max_ and Young's modulus, *E*_bend_) and external morphological parameters (primary feather rachis length, diameter and second moment of area at the calamus) in 180 primary feathers from four species of bird of differing flight style was investigated. Intraspecifically, both *E*_bend_ and *M*_max_ were strongly correlated with morphology, decreasing and increasing, respectively, with all three morphological measures. Without accounting for species, however, external morphology was a poor predictor of rachis structural properties, meaning that precise determination of aerial performance in extinct, feathered species from external rachis dimensions alone is not possible. Even if it were possible to calculate the second moment of area of the rachis, our data suggest that feather strength could still not be reliably estimated.

## 1. Background

To date, there is no consensus regarding the aerial performance of feathered dinosaurs [1–4]. It was recently proposed that one way to assess the flight capabilities of feathered dinosaurs is to use beam theory to estimate the strength of their flight feathers, but as acknowledged by the authors of the study, this requires some unvalidated assumptions to be made [5]. During gliding flight, in the absence of any dynamic forces on the wings, total lift forces will approximately equal body weight. These forces will increase dramatically during aerial manoeuvring and with wing flapping, necessitating more robust feathers. One of the fundamental assumptions of the approach that is yet to be validated is that medio-lateral rachis width, which is generally all that is available in terms of rachis cross-sectional biometrics on fossils, correlates strongly with the structural properties of the rachis. Furthermore, this correlation must be irrespective of species or feather position upon the wing as anyone investigating a particular feathered fossil has no extant reference species with which to compare feather characteristics directly.

The aerodynamic forces acting upon a feather are transmitted to the skeleton via the rachis, which acts as a tapered, cantilevered beam. The mechanical properties of beams subject to bending are primarily governed by their size, shape and orientation. In particular, key functional parameters such as rigidity (*EI*, N m^2^), maximum bending moment (*M*_max_, N m) and failure stress (*σ*_max_, N m^−2^) are strongly influenced by the distribution of material about the beam's neutral axis, the second moment of area (*I*, m^4^) [6]. In general, structures with larger values of *I* are more rigid than those with smaller values of *I* (assuming homogeneous material properties). *I* is not available for fossilized feathers, meaning that mechanical properties of the rachis may only be estimated from external morphological measures. Accordingly, Nudds & Dyke [5] estimated the buckling failure moment of primary feathers in *Archaeopteryx* and *Confuciusornis* from their rachis diameter (*Ø*_r_, mm) using Euler–Bernoulli beam theory. Relatively narrow *Ø*_r_ suggested that these species were not capable of the flapping flight seen in extant birds assuming similar hollow feather rachis morphology. Even when the feathers were modelled as solid beams, safety factors were much lower than in modern birds [5].

Previous cantilever testing of feather rachises has shown correlations between external morphological measures and structural properties [7,8]. *EI* calculated from the force required to deflect a feather by 6% of its length displays negative allometry with body mass, indicating relatively more flexible flight feathers in larger birds. Furthermore, feather rachis length from the distal tip of the rachis to the calamus (*l*_prim_, m) has been proposed to explain 63% of the variation in rachis stiffness [7]. Conversely, in blackcaps (*Sylvia atricapilla*), although *Ø*_r_ and feather mass explain much of the variation in mechanical performance, migratory individuals have stiffer rachises than sedentary individuals when controlling for these parameters [9]. Therefore, although differing flight demands may drive structural and material differences in feathers [10], rachis properties may not solely be reflected in external morphological measures [11]. Current knowledge regarding the structural properties of feather rachises is primarily based on cantilever tests, in which the feather is fixed at the calamus and its deflection measured under loading [7,8,12]. Although *EI* can be obtained using this method, the forces required for feather failure cannot. Furthermore, non-uniformity in cross-sectional shape and longitudinal curvature may introduce errors in the calculation of local stresses, which can be overcome by a three or four-point bending methodology [13]. Feather failure moments and their correlation with feather morphology are critical in any assessment of flight capabilities of extinct species and are currently lacking.

The forces that a feather shaft is able to withstand are determined by a combination of its material and structural properties. The rachis cortex is a complex hierarchical structure composed of microlayers of β-keratin, a composite material consisting of keratin fibres embedded within a viscoelastic matrix [14]. Within extant bird species, the material properties of β-keratin are poorly understood. A combination of indentation, tensile and compressive tests indicate that β-keratin hardness and Young's modulus values differ both within and between species, with Young's moduli varying from 0.045 to 10 GPa [12]. Some of this variation, however, may be due to methodological differences (e.g. indentation tests only provide data for the outer laminae of the feather rachis, whereas the results from tensile tests represent the summed material properties of the laminae). Structural and material testing of feathers has primarily focused on the outer primaries [7,15,16] or on primaries from single positions on the wing [12,17]. It is unclear to what extent feather material properties vary within individual feathers, with position along the wing or with flight style and species. Although *EI* has been shown to be similar at trailing, middle and leading portions of the wing within 13 species of bird [8], nanomechanical properties of the rachis vary inter- and intraspecifically and may be linked to differing flight styles [11].

In this study, the morphological and structural characteristics of feather rachises from four species of birds were measured using microscopy and three-point bending tests to test the hypothesis that Young's modulus and the maximum (failure point) bending moment of the rachis at the calamus can be predicted from external morphological measures alone (i.e. without reference to species).

## 2. Material and methods

### 2.1. Feathers

A total of 180 primary feathers were dissected from dried wings of four species of bird: carrion crow, *Corvus corone* (78 feathers); rock pigeon, *Columba livia* (29 feathers); mallard, *Anas platyrhynchos* (40 feathers); and common gull, *Larus canus canus* (33 feathers). In each species, the full range of primaries was represented, although some individuals had missing or damaged feathers. Humidity and hydration state affect the material properties of keratin [18,19]. Therefore, until testing, feathers were stored in an enclosed chamber above (but not in direct contact with) a saturated solution of potassium chloride, producing a relative humidity of 85%, which is a level commonly experienced by the test species in the UK [18].

### 2.2. Morphological measurements

**Figure 1.**
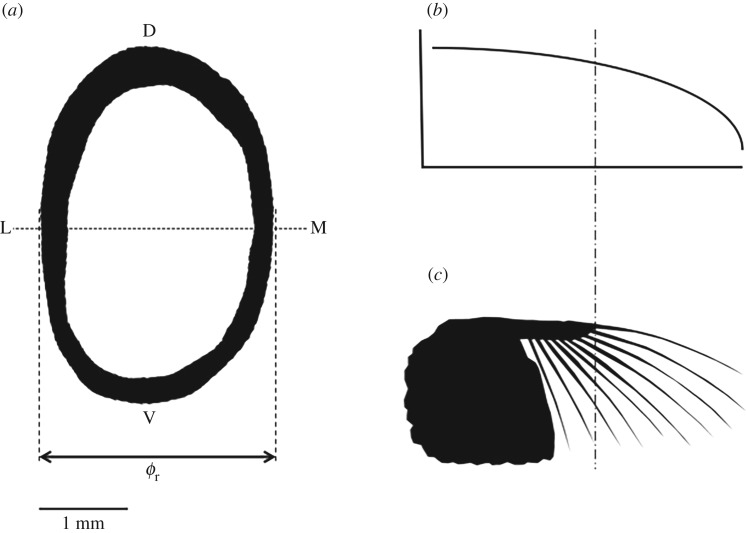
Typical cross section of a primary feather (crow right primary 7) at the calamus after image processing (*a*). The rachis second moment of area (mm^4^) was calculated about the medio-lateral (M-L) axis shown. Mean rachis cortical thickness (mm) was calculated over the entire black area representing the cortex. Rachis diameter (*Ø*_r_, mm) was the diameter at the widest point in the M-L direction. The assumed pattern of lift distribution along the wing, where the *x*-axis is distance along the wing (m) and the *y*-axis is lift (N) (*b*). An outline of a pigeon wing with the primary vanes removed (*c*). Lift acting on the primaries was calculated using the method of Nudds & Dyke [5] by dividing the lift acting on the outer portion of the wing (represented by the span from the insertion point of the primary, distal of the dotted and dashed line) by the number of primary feathers.

Prior to material testing, feathers were photographed and morphological measures of rachis length from the distal tip of the feather to the calamus at its point of insertion into the skin (*l*_prim_, mm) and medio-lateral diameter at the calamus at the same location (*Ø*_r_, mm) were taken. This location was chosen as it is where the bending moment acting on the feathers is highest. After material testing, the feathers were sectioned at the location where morphological measures were taken by using a Dremmel 400 series (Dremmel Europe, Breda, The Netherlands). These sections were imaged using a Leica MZ 9.5 with an IC D camera (Leica Microsystems, Wetzlar, Germany) and images were processed using the boneJ V 1.3.14 plugin [20] for ImageJ (v. 1.48 V, US National Institutes of Health, Bethesda, MD, USA (2014)) to obtain the second moment of area around the major axis (*I*_max_, mm^4^) and the mean cortical thickness (mm) (figure 1).

### 2.3. Three-point bending

The material properties of the rachis at the calamus were determined by subjecting feathers to quasi-static three-point bending in the dorsoventral plane (support distance = 25 mm, extension speed = 5 mm min^−1^). Dorsal loading of the rachis mimics the natural loading of the feather during the downstroke, where compressive and tensile forces on the dorsal and ventral surfaces, respectively, are highest [13]. Force and displacement at the calamus were measured using a Houndsfield test machine fitted with a 100 N load cell. The resulting force displacement curves were used to calculate the maximum bending moment (*M*_max_, N m) using the standard equation for a beam in three-point bending:

2.1 $$M_{\max}=\frac{F_{\max}L}{4},$$

where *F*_max_ is maximum force prior to failure (N, where failure was defined as the point at which the force–displacement slope first became negative) and *L* is the distance between the sample supports (m). Even though the rachis is a bio-composite material, in order to simplify the calculation, it was assumed that it was a homogeneous isotropic material. Young's modulus (*E*_bend_, GPa) was calculated using

2.2 $$E_{\mathrm{bend}}=\frac{FL^{3}}{48\delta I},$$

where *F* is the change in force (N) and *δ* is the displacement (m) of the sample over the initial portion of the force–displacement curve.

### 2.4. Predictive models

The relationships between rachis structural properties and morphological measures were determined using ordinary least-squares regressions. The predictive intervals of these regressions were used to assess the viability of morphology alone as a predictor of feather structural properties without knowledge of species assignment (which is clearly the case with feathered fossils). Ordinary least-squares regressions were used rather than reduced major axis regressions as the errors of our morphological measurements are likely to be lower than those in the calculations of feather structural properties.

### 2.5. Predicting feather safety factors

To determine whether *Ø*_r_ could be used to estimate the mechanical performance of flight feathers in a biologically relevant context, feather rachis safety factors (maximum bending moments/estimated moments resulting from lift) were estimated using the method of Nudds & Dyke [5]. The lift on one wing was assumed to equal half the body weight (obtained from Dunning [21]) and to follow an elliptical lift distribution. The force acting on a single feather (*F*_f,_ *N*) was then calculated by dividing the lift acting on the portion of the wing from the base of the outermost primary to the wingtip by the number of primaries. Measures of wingspan from the base of the outermost primary were performed on dried wings prior to feather extraction and thus subject to error. However, resulting errors in our estimations of lift forces acting on the primaries would not compromise our evaluation of the predictive capabilities of rachis morphology. The span measured from the base of the outermost primaries is smaller than the true outer wingspan, measured from the base of the innermost primary. Both under- or overestimations of span from the base of the outermost primaries would therefore result in overestimates of rachis safety factors. The larger span was not used in order to make our data comparable with those of Nudds & Dyke [5]. Moments acting upon individual primary feathers (*M*_f_, *N*m) were estimated assuming an elliptical lift distribution using

2.3 $$M_{f}=F_{f}\left(\frac{4l_{\mathrm{prim}}}{3\pi }\right).$$

An absence of data regarding the moments acting upon individual flight feathers means that these values are only approximations but are sufficient in order to evaluate the predictive power of morphological measures. Maximum (SF_max_) and minimum (SF_min_) safety factors were calculated from the upper and lower predictive bounds of the regression of *M*_max_ with *Ø*_r_, respectively. Safety factors were also estimated from the regression line itself (SF_reg_) and from our measured values of *M*_max_ (SF_measured_).

### 2.6. Data analyses

Analysis of covariance (ANCOVA) was used to identify species-specific differences in rachis structural properties across the range of each morphological measure. If the interaction term (species × morphological measure) was non-significant indicating similar slopes, they were removed from the ANCOVA model and the test was rerun assuming parallel slopes (testing for differences in the intercepts only). A Tukey's *post hoc* test was used to determine any individual differences in the intercepts where slopes were common. One-way ANOVA was used to identify differences in safety factors between species, and *t*-tests were used to determine differences between measured and estimated safety factors within species. Regressions and ANOVAs were carried out in R v. 3.1.2 [22], *t*-tests were carried out in SPSS v. 22 (IBM, Somers, NY, USA) and ANCOVAs were conducted in Matlab R2013a using the native ANCOVA tool (The MathWorks, Inc., Natick, MA, USA). Safety factors are presented with ±s.e.

## 3. Results

### 3.1. *E*_bend_

**Figure 2.**
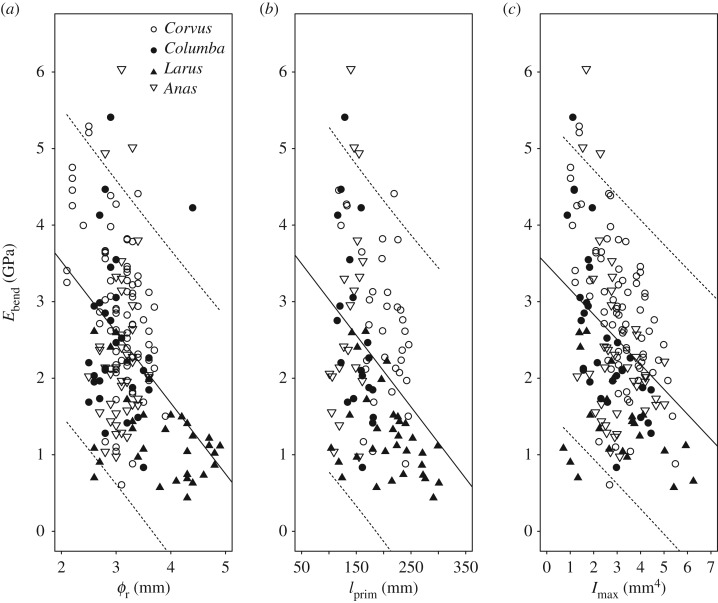
Linear regressions of Young's modulus (*E*_bend_, GPa) with (*a*) rachis diameter (*Ø*_r_, mm) (*E*_bend_ = −0.93. *Ø*_r_ + 5.38), (*b*) rachis length (*l*_prim_, mm) *(E*_bend_ = −0.0094. *l*_prim_ + 3.96) and (*c*) rachis second moment of area (*I*_max,_ mm^4^) (*E*_bend_ = −0.33.*I*_max_ + 3.48) in four species of extant birds (carrion crow, *Corvus corone*; rock pigeon, *Columba livia*; common gull, *Larus canus canus*; mallard, *Anas platyrhynchos*). Dashed lines indicate the upper and lower predictive limits of the linear regression.

There was a negative relationship between *E*_bend_ and *Ø*_r_ (figure 2*a* and table 1; *p* < 0.001), *l*_prim_ (figure 2*b* and table 1; *p* < 0.01) and *I*_max_ (figure 2*c* and table 1; *p* < 0.001). The incremental change in *E*_bend_ differed between species when plotted against *Ø*_r_ (figure 2*a* and table 1; *p* < 0.001), *l*_prim_ (figure 2*b* and table 1; *p* < 0.001) and *I*_max_ (figure 2*c* and table 1; *p* < 0.001).

**Table 1.** Summary of the ANCOVA analysis for the structural properties of feather rachises with morphological measures. *E*_bend_ was measured in gigapascal. *M*_max_ was measured in newton metre.

		full model			
dependent variable	source	d.f.	*r*^2^	*F*	*p*-value
*E*_bend_	species	3	0.14	10.91	0.0000
	*Ø*_r_	1	0.06	12.75	0.0005
	species × *Ø*_r_	3	0.05	3.58	0.0151
	error	171			
*E*_bend_	species	3	0.24	13.62	0.0000
	*l*_prim_	1	0.05	7.65	0.0068
	species × *l*_prim_	3	0.13	7.05	0.0002
	error	99			
*E*_bend_	species	3	0.16	15.63	0.0000
	*I*_max_	1	0.16	46.57	0.0000
	species × *I*_max_	3	0.11	11.38	0.0000
	error	172			
*M*_max_	species	3	0.38	66.91	0.0000
	*Ø*_r_	1	0.28	146.51	0.0000
	species × *Ø*_r_	3	0.03	4.83	0.0030
	error	171			
*M*_max_	species	3	0.31	59.59	0.0000
	*l*_prim_	1	0.48	274.01	0.0000
	species × *l*_prim_	3	0.03	6.03	0.0008
	error	99			
*M*_max_	species	3	0.34	61.97	0.0000
	*I*_max_	1	0.32	173.76	0.0000
	species × *I*_max_	3	0.02	3.15	0.0263
	error	172			

### 3.2. *M*_max_

**Figure 3.**
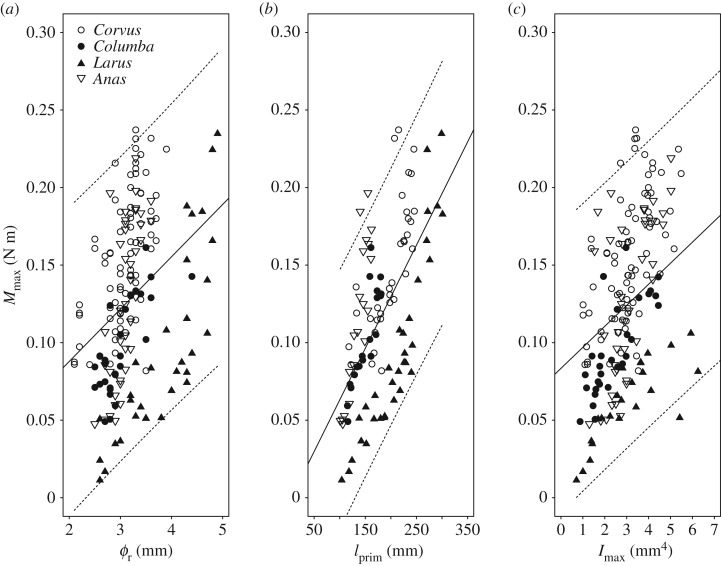
Linear regressions of the maximum bending moment (*M*_max_, N m) with (*a*) rachis diameter (*Ø*_r_, mm) (*M*_max_ = 0.034. *Ø*_r_ + 0.0204), (*b*) rachis length (*l*_prim_, mm) (*M*_max_ = 0.00067.*l*_prim_ – 0.0035) and (*c*) rachis second moment of area (*I*_max_, mm^4^) (*M*_max_ = 0.014.*I*_max_ + 0.08) in four species of extant birds (carrion crow, *Corvus corone*; rock pigeon, *Columba livia*; common gull, *Larus canus canus*; and mallard, *Anas platyrhynchos*). Dashed lines indicate the upper and lower predictive limits of the linear regression.

There was a positive relationship between *M*_max_ and *Ø*_r_ (figure 3*a* and table 1; *p* < 0.001), *f*_prim_ (figure 3*b* and table 1; *p* < 0.001) and *I*_max_ (figure 3*c* and table 1; *p* < 0.001). The incremental change in *M*_max_ differed between species when plotted against *Ø*_r_ (figure 3*a*, *p* < 0.01), *l*_prim_ (figure 3*b* and table 1; *p* < 0.001) and *I*_max_ (figure 3*c* and table 1; *p* < 0.05).

### 3.3. Safety factors

**Figure 4.**
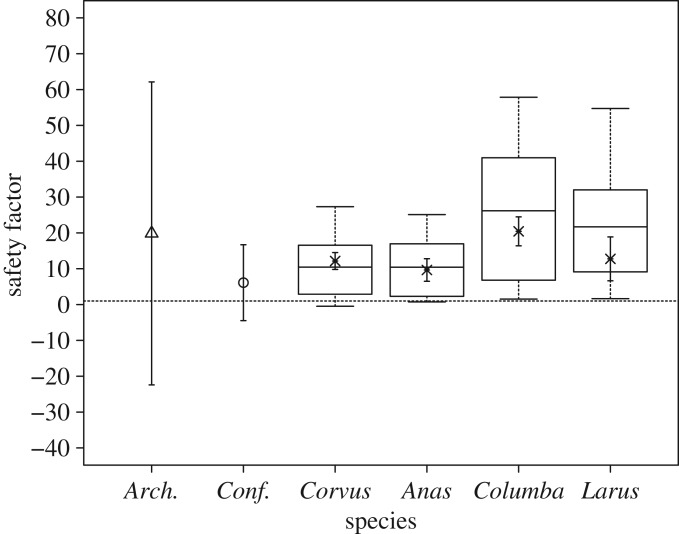
Box plot of estimated primary feather rachis safety factors for the four species of extant birds tested, based on the maximum and minimum values of the maximum bending moment (*M*_max_, N m) estimated from the upper and lower predictive bounds of the regression of *M*_max_ with rachis diameter (*Ø*_r_, mm), respectively. Dark lines in boxes represent median values. Crosses indicate safety factors estimated from actual values of *M*_max_ (_­_±s.d.). Estimated rachis safety factors for *Archaeopteryx* (*Arch.*, triangle) and *Confuciusornis* (*Conf.*, circle) using the data from Nudds & Dyke [5] are included for comparison (error bars represent the upper and lower predicted safety factors). The dashed horizontal line represents a safety factor of 1.

SF_measured_ was significantly different between species (figure 4: One-way ANOVA; *F*_3,102_ = 24.64 *p* < 0.001). A Tukey's HSD *post hoc* test revealed no measurable differences between estimates for crows (mean = 1216 ± 40%), gulls (mean = 1275 ± 108%) or mallards (mean = 965 ± 70%). Pigeon estimates were significantly higher than those of the other species tested (mean = 2043 ± 90%). Within each species, SF_reg_ and SF_measured_ were significantly different with the exception of the mallard (*t*-test, *p* = 0.22).

## 4. Discussion

The data presented here clearly demonstrate that accurate determination of aerial performance in extinct, feathered species from external rachis dimensions alone is not possible. Predicting the forces feathers are able to sustain based on external rachis morphology relies on the assumption that *M*_max_ increases predictably with increasing *Ø*_r_ (and therefore *I*). Variation in the relationship between *M*_max_ and *Ø*_r_, and *l*_prim_ is low within species, giving *Ø*_r_ and *l*_prim_ relatively good predictive power over *M*_max_. However, there is significant interspecific variation, diminishing the accuracy of predictions of *M*_max_ made from external morphological measures, which in turn limits the accuracy of estimates of feather safety factors (figure 4). Species-specific measured values of *M*_max_ yield mean safety factors of between 9.65 ± 3.15 (mallard mean ± s.d.) and 20.43 ± 4.04 (pigeon mean ± s.d.), which are close to the range expected for birds capable of flapping flight, thus validating our methodology. For example, Corning & Biewener [13] estimated similar safety factors of between 9 and 17 in the primary feathers of flapping pigeons. However, predictions based upon the lower and upper predictive limits of the dataset yield, respectively, a range of safety factors from −0.47 (crow outermost primary lower estimate) to 58 (pigeon innermost primary upper estimate). Similarly, in three of the four species tested, SF_reg_ and SF_measured_ differed. Clearly, feather failure properties cannot be predicted without a prior knowledge of the species-specific relationship between *M*_max_ and morphological measures. As a result, using the method and rachis measurements of Nudds & Dyke [5], with the data gathered here, the safety factors range from −22.4 to 62.1 in *Archaeopteryx* and from −4.5 to 16.7 in *Confuciusornis* (figure 4). This range spans the full complement of potential flight abilities. Of course, these safety factors are heavily reliant on the estimated body masses of these extinct species. Halving the body mass estimates would approximately double the mean safety factor estimates and, of course, doubling the body mass estimates would approximately halve the mean safety factor estimates. Irrespective of the mass estimates, however, the range of safety factors and lack of predictive power would remain. The fact that body mass estimates are also subject to errors compounds further the ability to assess flight capabilities from flight feather rachis morphology [23,24]. It should also be noted that depending on the wing, the assumption that the lift forces distal of the base of the outermost primary is the total supported by the primaries [5] is an underestimate, because not all of the area occupied by the primaries is encompassed (figure 1). The effect of this would be to elevate the safety factors.

Although *M*_max_ determines the failure properties of the flight feathers, *EI* is also a critical determinant of flight ability. Feathers with a high *M*_max_ but low stiffness would be of little use during either gliding or flapping. Indeed, high safety factors of feathers are suggested as representing an exaptation of selection for high stiffness and low weight rather than adaptation towards high bending strength [13]. Although *E*_bend_ showed a significant relationship with external rachis morphology within species, high interspecific variability eroded the predictive power of *Ø*_r_ and *l*_prim_. Variability was such that the direction of the incremental change in *E*_bend_ with *l*_prim_ differed between species, decreasing in the crow, pigeon and gull but increasing in the mallard. Although *E*_bend_ decreased with increasing *Ø*_r_, the rigidity of feathers, *EI* increased as a result of higher *I* at larger rachis diameters. Therefore, feathers with wider rachises are both stronger and resist deflection more than those with narrow rachises. Measured values of *EI* are consistent with cantilever tests previously performed by Worcester [8].

The predicted values of *M*_max_ used by Nudds & Dyke [5] to estimate safety factors in *Archaeopteryx* and *Confuciusornis* were calculated using

**Figure 5.**
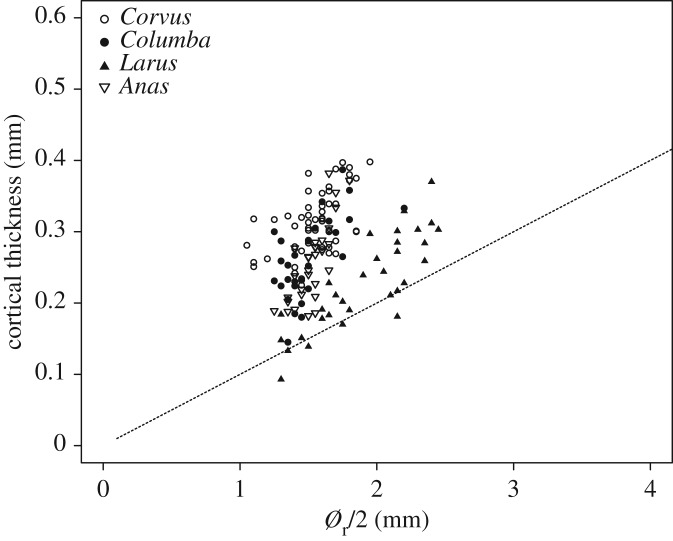
Mean rachis cortical thickness (mm) plotted against rachis radius (*Ø*_r_/2, mm) in four species of extant birds (carrion crow, *Corvus corone*; rock pigeon, *Columba livia*; mallard, *Anas platyrhynchos*; common gull, *Larus canus canus*). The dashed line represents a wall thickness of less than 10% of rachis radius, below which the rachis may be defined as a thin-walled cylinder.

4.1 $$M=\frac{KErt^{2}}{1-v^{2}},$$

where *K* is a constant (≈ 1), *E* is Young's modulus of feather keratin, *r* is the mean radius of the rachis cross section, *t* is mean wall thickness and *ν* is Poisson's ratio. The accuracy of this equation relies on accurate estimates of *E* and *ν*, as well as the assumptions that (i) lift distribution is elliptical across the span and the forces distally are equally distributed across the primaries and (ii) the feather rachis at the calamus is a thin-walled cylinder (in which cortex thickness is no more than 10% of the cylinder's radius) and is isotropic. Reported material properties of β-keratin are highly variable in the literature, varying within and between species [15,17,25]. This variability is likely, because flight feather rachises are not isotropic but consist of multiple layers of differentially oriented keratin fibres [26,27], primarily longitudinally oriented, meaning transverse stiffness is less than longitudinal stiffness, making the shaft prone to flattening [6]. Fibre orientation also varies across species of diverse phylogenies and/or flight styles [11]. The data presented here indicate that flight feathers are not thin-walled, with less than 3% of feathers (five feathers from the gull) meeting the required criteria (figure 5). Whereas thin-walled cylinders are prone to failure by local buckling, thick-walled cylinders fail through a combination of buckling and tensile failure. Nonetheless, our feathers primarily failed through local buckling as was found in pigeon primary feather shafts [13]. As a result, our measured values of *M*_max_ are broadly in agreement with the predictions of Nudds & Dyke [5]. Crucially, however, these predictions fail to account for the observed variability due to deviations from the model's simplistic assumptions. These nuances in material and structural properties are likely to be adaptations to the differing flight styles and life-history traits of the birds tested. For example, gulls are active soaring species with high aspect ratio wings, whereas crows have rounded, intermediate aspect ratio wings suited to bursts of high-speed flapping [28,29]. The differing mechanical demands upon the feathers of these species are likely to be driving the differences in the observed relationships between structural properties and morphology. Indeed, flight style does appear to affect feather morphology [10]. Therefore, if we are to determine the flight capabilities of extinct birds from morphology-based, predicted structural properties, we must first have an idea of their flight styles, thus creating a circular argument. It is unclear if flight style can be estimated for extinct species from measures of wing and primary feather morphology [30,31]. Any such predictions also rely on the assumption that feather barb geometries were similar in extinct species. This assumption is not necessarily supported as barb geometries in Mezozoic taxa stem-ward of the Ornithurae and Enantiornithes differ from those of modern birds [32].

It is unsurprising that external measures such as *Ø*_r_ and *l*_prim_ are not predictive of rachis structural properties, as they do not describe the distribution of material within that structure. This is particularly true when comparing rachises, which are often oval as opposed to circular in cross section at their base. The distribution of material relative to a beam's neutral axis (*I*_max_) is critical to its flexural stiffness. Higher *I*_max_ is associated with an increased rigidity and is highest at the calamus in feathers [12]. However, despite the intimate relationship between *I*_max_ and beam structural properties, *I*_max_ predicted neither *M*_max,_ nor *E*_bend_ very well. Again this resulted from interspecific differences in the values of *M*_max_ and *E*_bend_ at any given value of *I*_max_ despite strong associations within species.

Wang *et al.* [7], using cantilever tests, showed a strong relationship between the force required to deflect feathers by 6% of *l*_prim_ and the morphological measures of dorsoventral *Ø*_r_ taken 75% along the length of the rachis (*r*^2^ = 0.92) and *l*_prim_ (*r*^2^ = 0.89). Although the data from this study are not directly comparable to ours due to methodological differences, the high coefficients of determination are not in keeping with the present findings. For example, here, the relationship between *E*_bend_ and *I*_max_, perhaps the best candidate for a morphological predictor of rachis structural properties, was variable between species, resulting in an *r*^2^ of just 0.26 overall. Here we measured *E*_bend_ for every primary feather from both wings of multiple individuals for each of the four species tested (body mass range = 0.35–1.08 kg), thus capturing the full scope for variation in *E*_bend_ in these species across a narrow range of *Ø*_r_. Conversely, Wang *et al*. [7] obtained single feather measures (the longest primary) from 35 bird species (body mass range = 0.02–8.91 kg). Although such data are useful in demonstrating the broad relationships in rachis properties across bird species, it does not elucidate the variation within and between species required to establish the accuracy of any predictions of the structural properties made for any specific value of *Ø*_r_.

The mechanical performance of a feather is dictated by a combination of the structural and material properties of its rachis. The absence here of any gross structural predictor of rachis mechanical properties, therefore, suggests variation in the material properties and microstructure of the rachis across species. Avian β-keratin is a composite material comprising a viscoelastic matrix with embedded fibres. Alterations to either of these components can influence the material properties of the rachis. For example, Cameron *et al.* [17] showed that Young's modulus along the rachis varies with differential orientation of keratin molecules, from a disordered state at the calamus to a more ordered configuration, associated with increased Young's modulus. Although β-keratin appears to be biochemically conserved [33,34], it is unclear as to whether the keratin of extinct feathered species was similar to that of modern birds. The accuracy of any predictions regarding the structural properties of ancient feathers will also be weak until we have fully categorized β-keratin in extant integumentary organs. Hence, despite the theoretical approach used in the study of Nudds & Dyke [5], providing realistic values in terms of the safety factors calculated for the modern birds used as controls in the study, it is compromised by the assumption that feather rachises are homogeneous structures across species.

## 5. Conclusion

In conclusion, there are interspecific differences in the relationships between morphological measures and the structural properties of feather rachises at the calamus. This variability is likely to result from microstructural and material variations within the rachis, which may serve as adaptations to different flight styles. As a result, it is presently impossible to determine the structural properties of ancient feathers and, in turn, the flight capabilities of extinct feathered species from their feather morphology. More accurate predictions of flight ability in proto-birds first require a deeper understanding of how the mechanical demands of flight in modern birds have driven the observed variability in feathers and their keratin.

## Supplementary Material

Raw rachis data: Raw rachis morphological and structural properties used in our analysis

## Data accessibility

The data used for the analyses are available as the electronic supplementary material.
